# Supplementary material for: Cornflower Extract and Its Active Components Alleviate Dexamethasone-Induced Muscle Wasting by Targeting Cannabinoid Receptors and Modulating Gut Microbiota
Source: Nutrients. 2024 Apr 11;16(8):1130. doi: 10.3390/nu16081130 (PMC11054969; doi:10.3390/nu16081130)
Supplement: Supplementary file 1 [file nutrients-16-01130-s001.zip › nutrients-2875505-supplementary.pdf]

Supplementary Materials

List of Figures and Table

Table S1. HPLC running method

Figure S1. (A) ^{1}H-NMR spectrum of compound **1** in CH_3OH-d_4 at 400 MHz. (B) ^{13}C-NMR spectrum of compound **1** in CH_3OH-d_4 at 100 MHz

Figure S2. (A) ^{1}H-NMR spectrum of compound **2** in CH_3OH-d_4 at 500 MHz. (B) ^{13}C-NMR spectrum of compound **2** in CH_3OH-d_4 at 125 MHz

Figure S3. (A) ^{1}H-NMR spectrum of compound **3** in CH_3OH-d_4 at 500 MHz. (B) ^{13}C-NMR spectrum of compound **3** in CH_3OH-d_4 at 125 MHz.

Figure S4. (A) ^{1}H-NMR spectrum of compound **4** in CH_3OH-d_4 at 500 MHz. (B) ^{13}C-NMR spectrum of compound **4** in CH_3OH-d_4 at 125 MHz.

Figure S5. (A) ^{1}H-NMR spectrum of compound **5** in DMSO-d_6 at 500 MHz. (B) ^{13}C-NMR spectrum of compound **5** in DMSO-d_6 at 125 MHz

Figure S6. (A) ^{1}H-NMR spectrum of compound **6** in CH_3OH-d_4 at 500 MHz. (B) ^{13}C-NMR spectrum of compound **6** in CH_3OH-d_4 at 125 MHz

Figure S7. (A) ^{1}H-NMR spectrum of compound **7** in CH_3OH-d_4 at 500 MHz. (B) ^{13}C-NMR spectrum of compound **7** in CH_3OH-d_4 at 125 MHz

Figure S8. (A) ^{1}H-NMR spectrum of compound **8** in CH_3OH-d_4 at 500 MHz. (B) ^{13}C-NMR spectrum of compound **8** in CH_3OH-d_4 at 125 MHz

Figure S9. (A) ^{1}H-NMR spectrum of compound **9** in DMSO-d_6 at 500 MHz. (B) ^{13}C-NMR spectrum of compound **9** in DMSO-d_6 at 125 MHz

Table S1. HPLC running method

	Time	ACN (0.1% FA)	Water (0.1% FA)
1	0.0	5.0	95.0
2	17.0	20.0	80.0
3	25.0	30.0	70.0
4	32.0	100.0	00.0
5	35.0	100.0	00.0
6	37.0	5.0	95.0
7	38.0	5.0	95.0

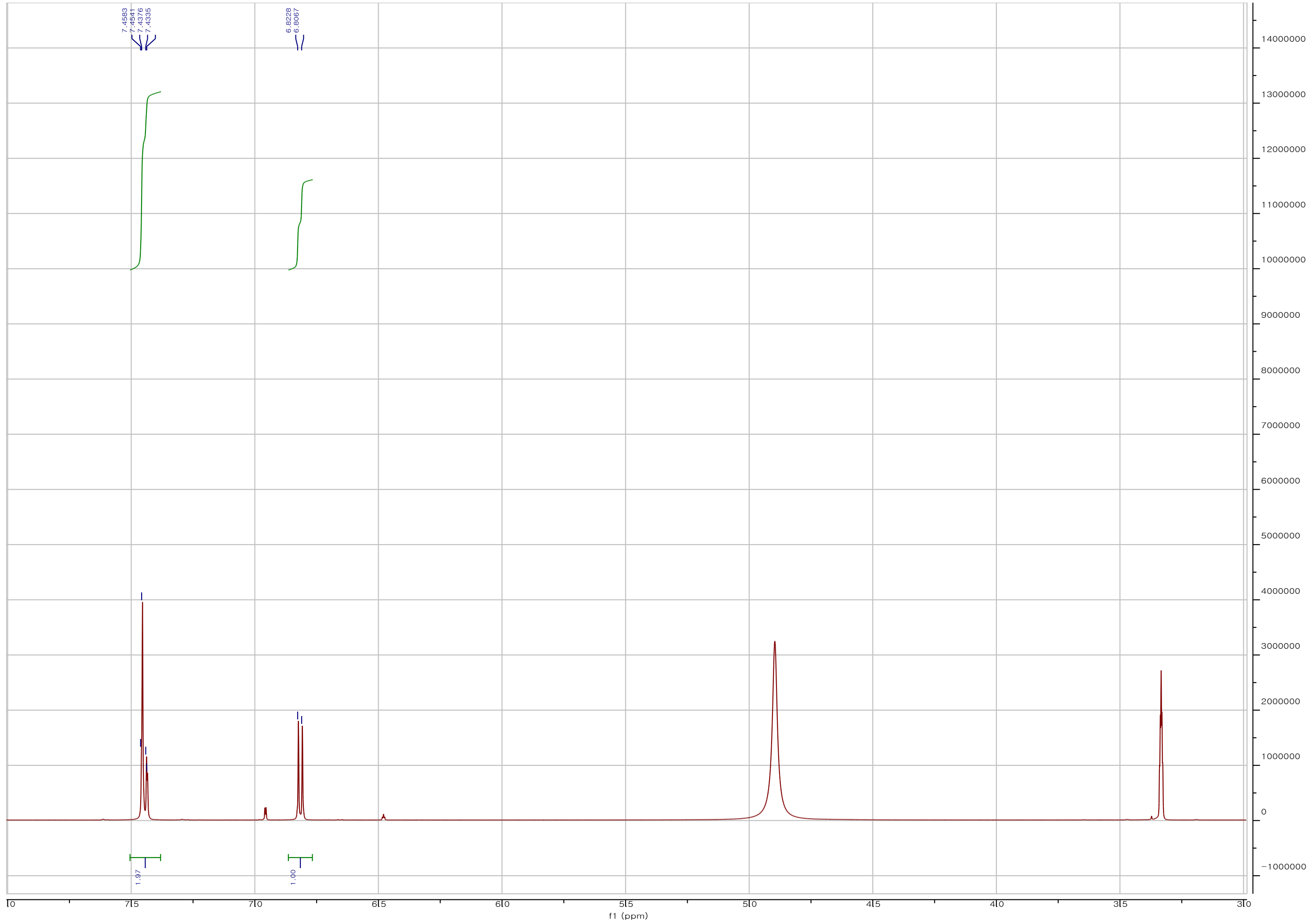

(A)

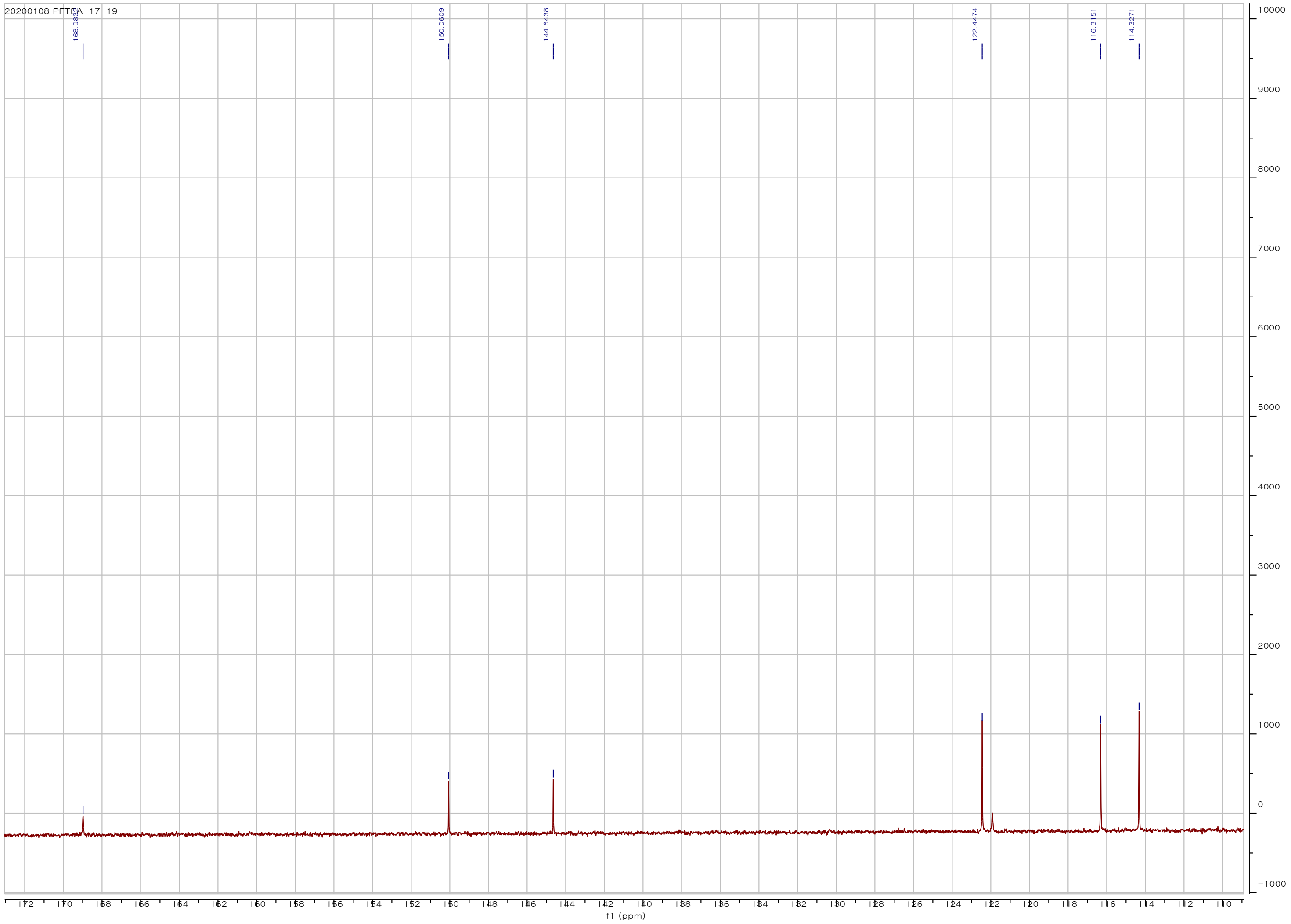

(B)

Figure S1. (A) ^{1}H-NMR spectrum of compound **1** in CH_3OH-d_4 at 400 MHz. (B) ^{13}C-NMR spectrum of compound **1** in CH_3OH-d_4 at 100 MHz.

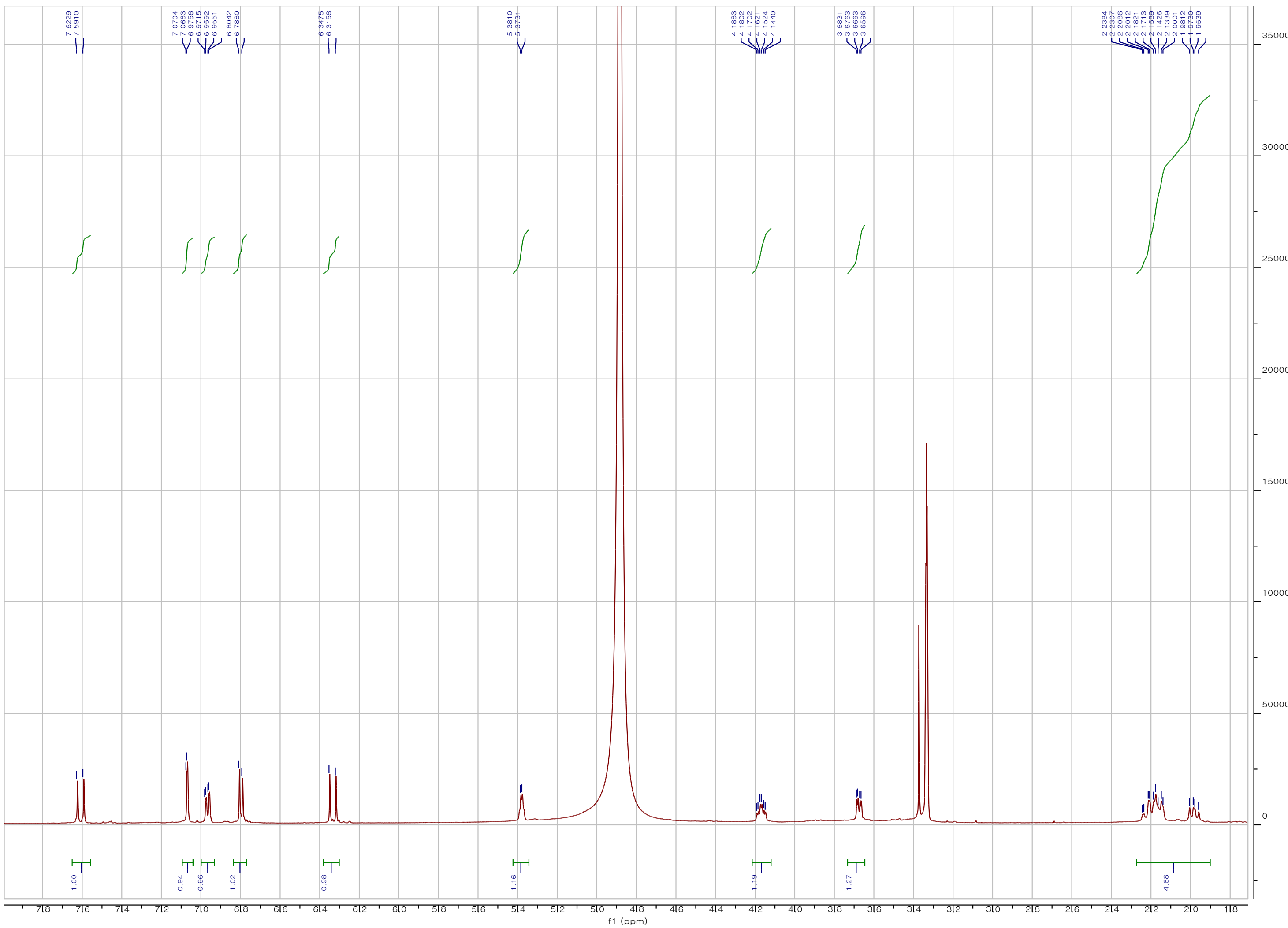

7.6229
7.5910
7.0704
7.0663
6.9756
6.9715
6.9592
6.9551
6.8042
6.7980
6.3475
6.3158
5.3810
5.3731
4.1883
4.1802
4.1702
4.1621
4.1524
4.1440
3.6831
3.6763
3.6663
3.6596
2.2384
2.2307
2.2098
2.2012
2.1821
2.1713
2.1589
2.1426
2.1339
2.0001
1.9812
1.9730
1.9539
1.00
0.94
0.96
1.02
0.98
1.16
1.19
1.27
4.68
f1 (ppm)

(A)

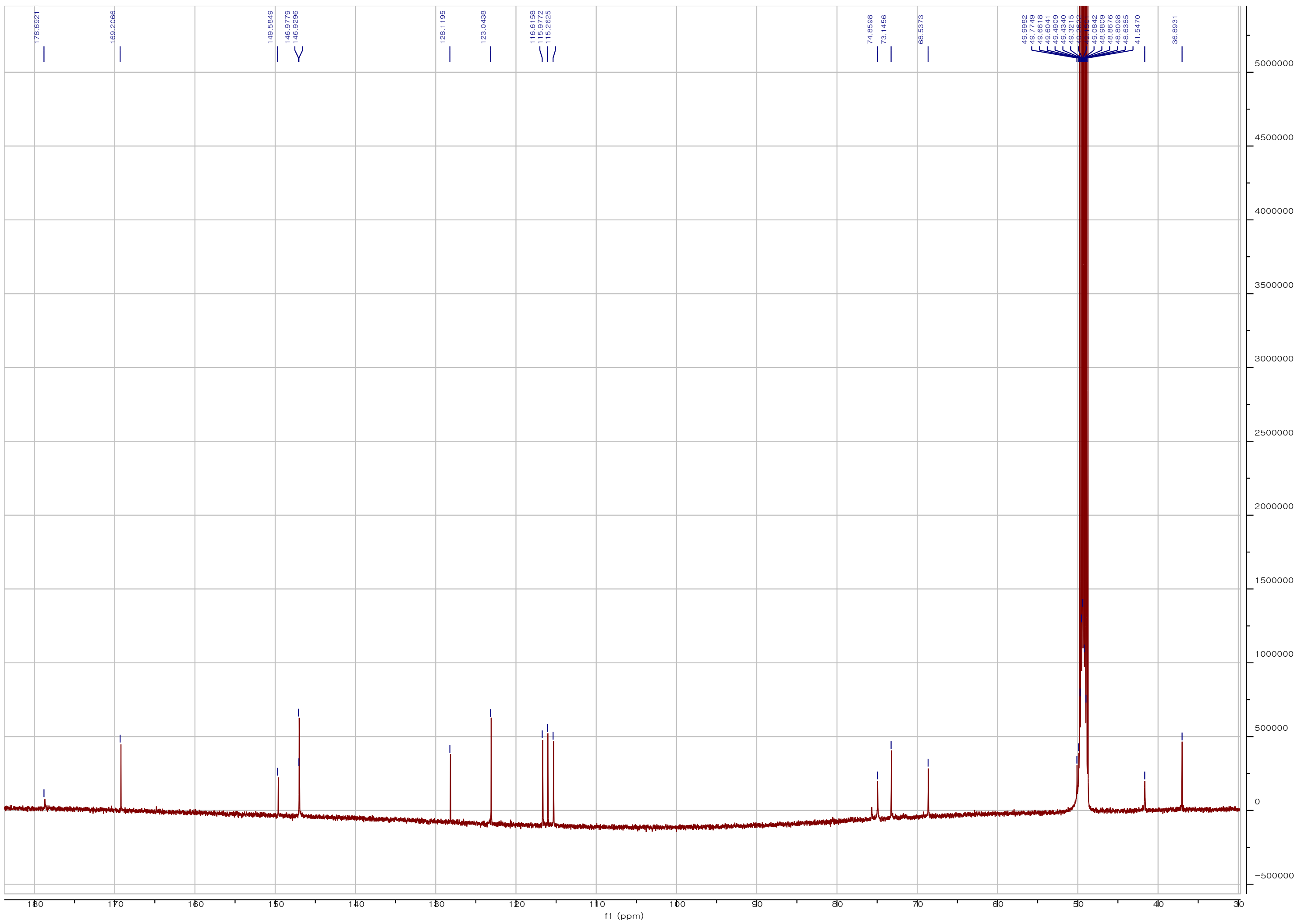

(B)

Figure S2. (A) ^{1}H-NMR spectrum of compound **2** in CH_3OH-d_4 at 400 MHz. (B) ^{13}C-NMR spectrum of compound **2** in CH_3OH-d_4 at 100 MHz.

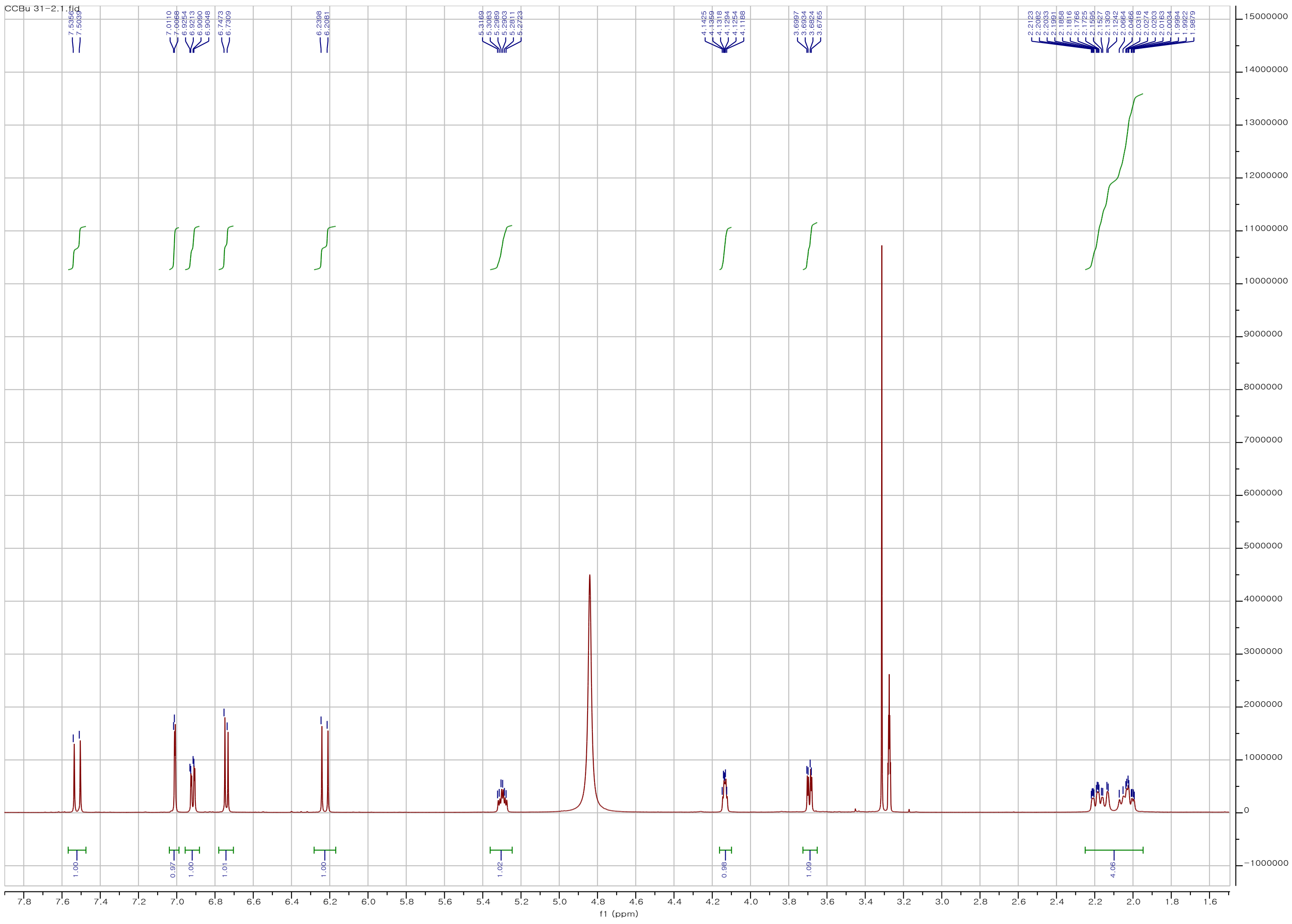

(A)

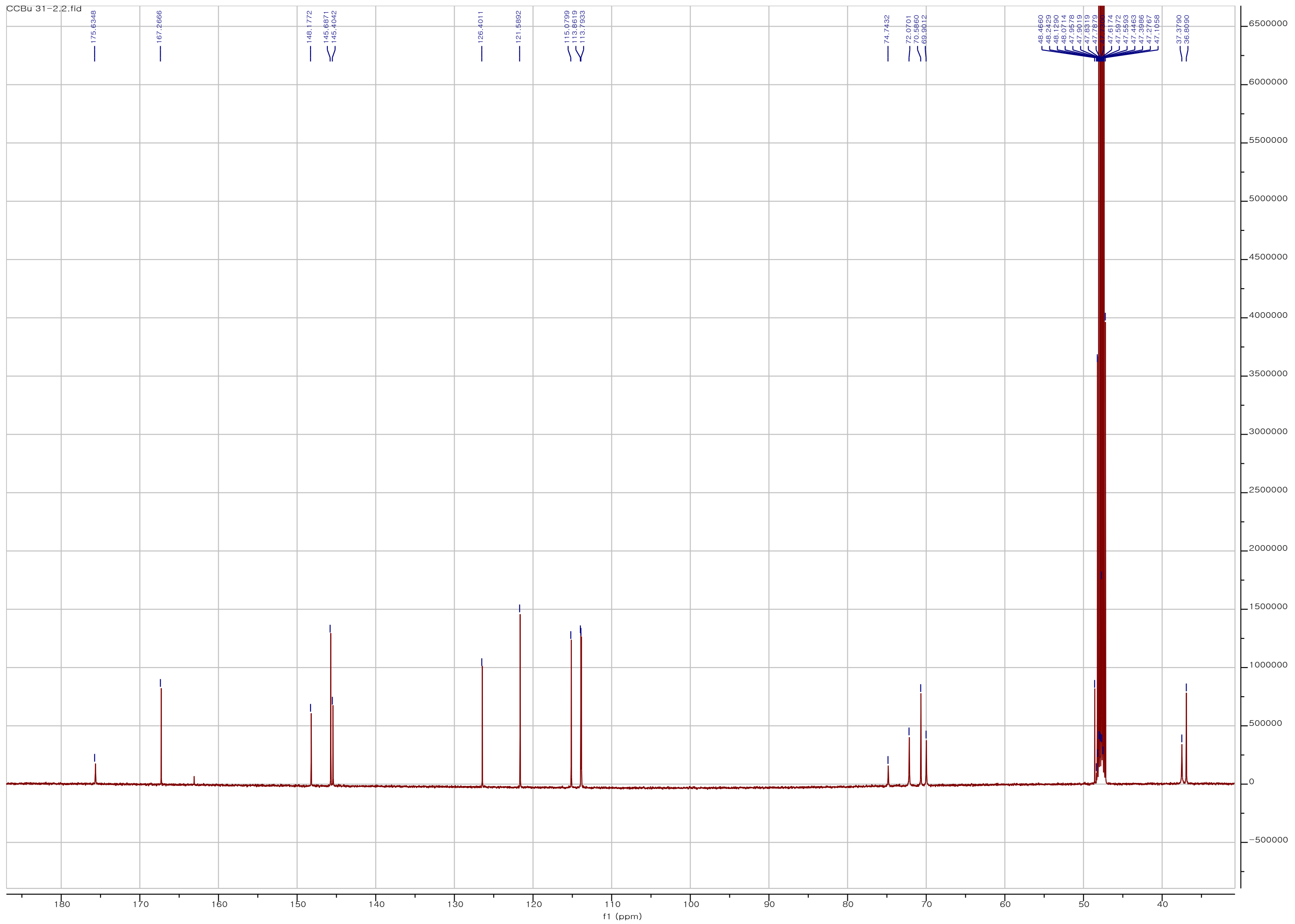

(B)

Figure S3. (A) ^{1}H-NMR spectrum of compound **2** in CH_3OH-d_4 at 400 MHz. (B) ^{13}C-NMR spectrum of compound **2** in CH_3OH-d_4 at 100 MHz.

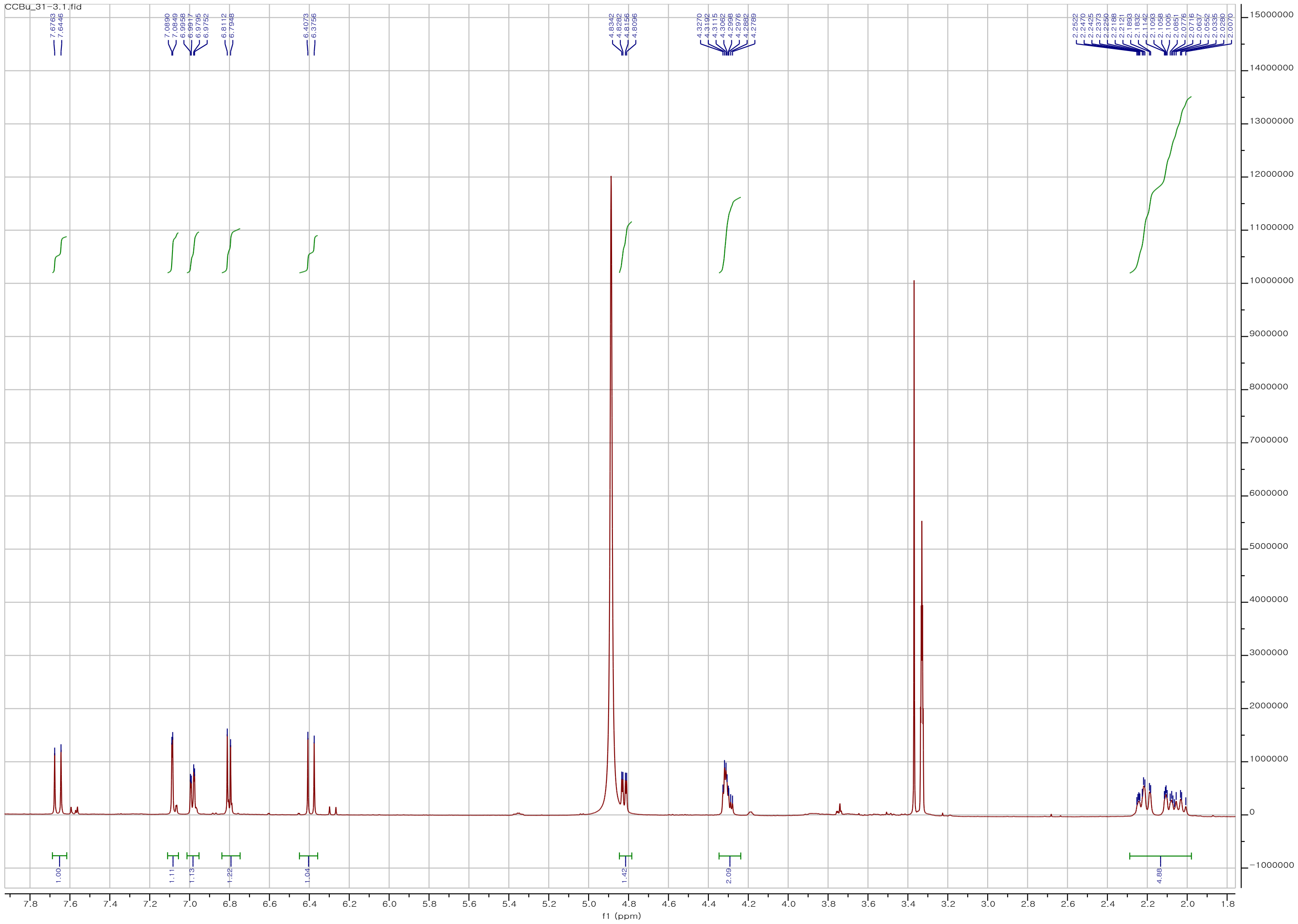

(A)

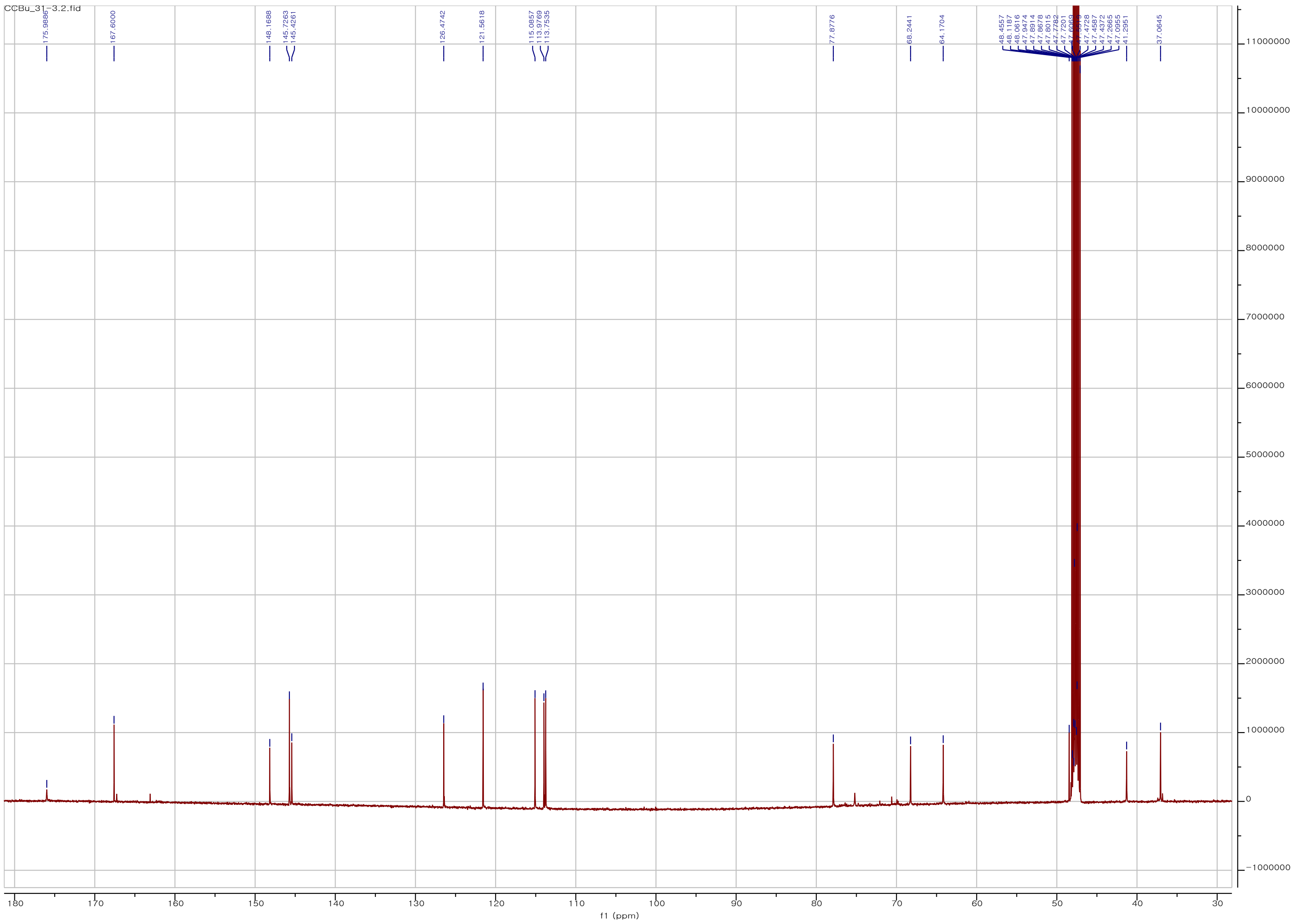

(B)

Figure S4. (A) ^{1}H-NMR spectrum of compound **4** in CH_3OH-d_4 at 400 MHz. (B) ^{13}C-NMR spectrum of compound **4** in CH_3OH-d_4 at 100 MHz.

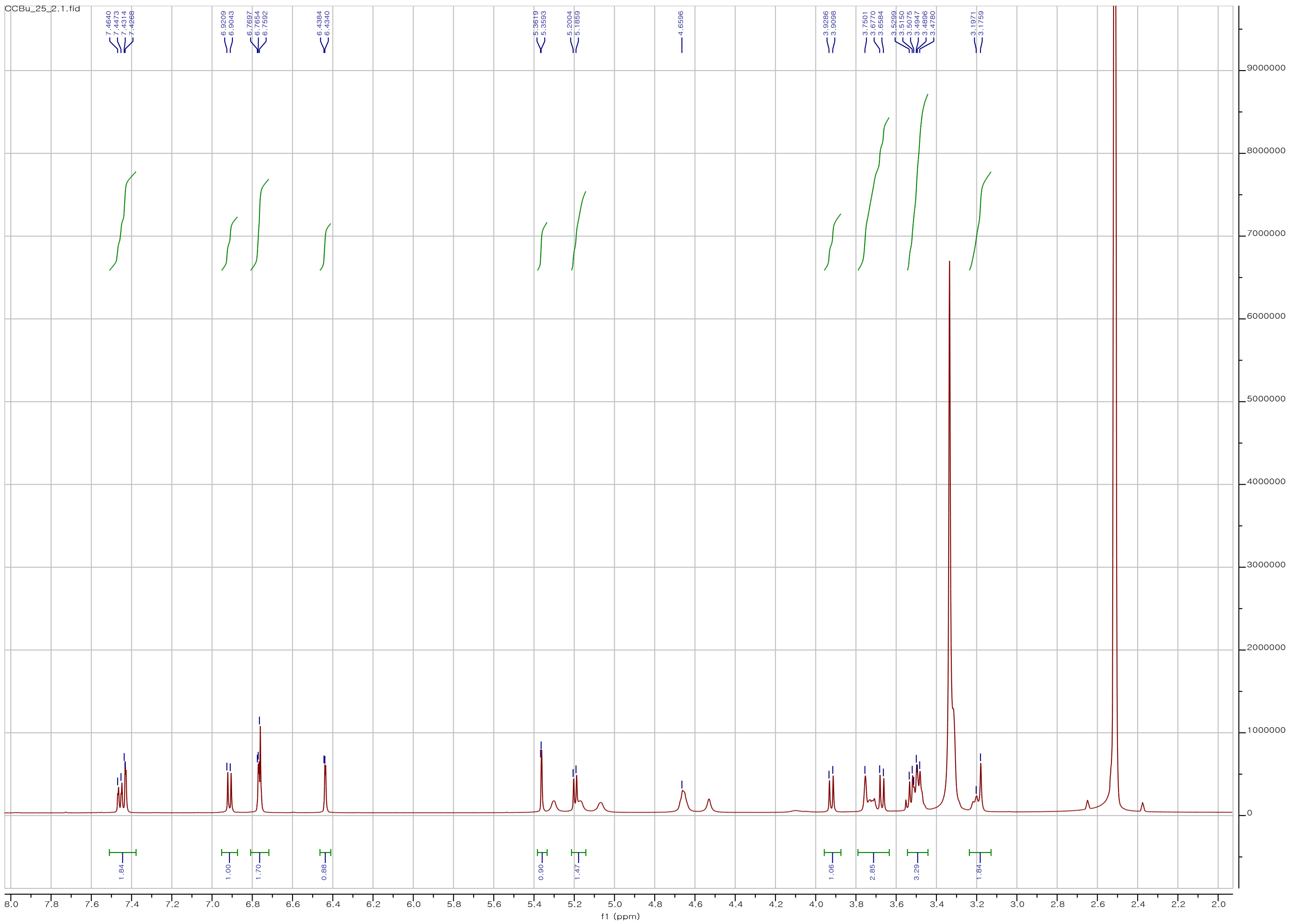

(A)

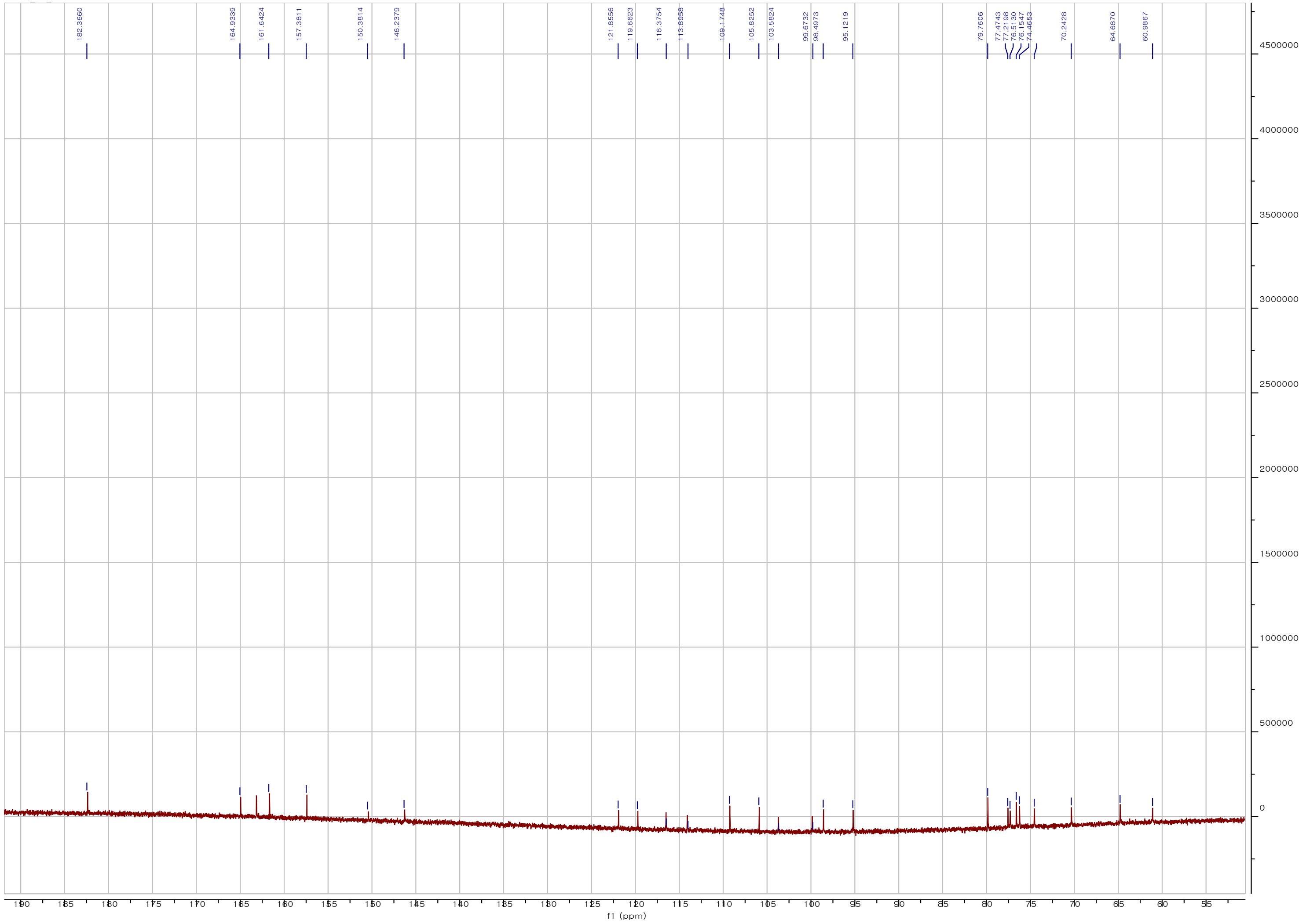

(B)

Figure S5. (A) ^{1}H-NMR spectrum of compound **5** in DMSO-d_6 at 500 MHz. (B) ^{13}C-NMR spectrum of compound **5** in DMSO-d_6 at 125 MHz.

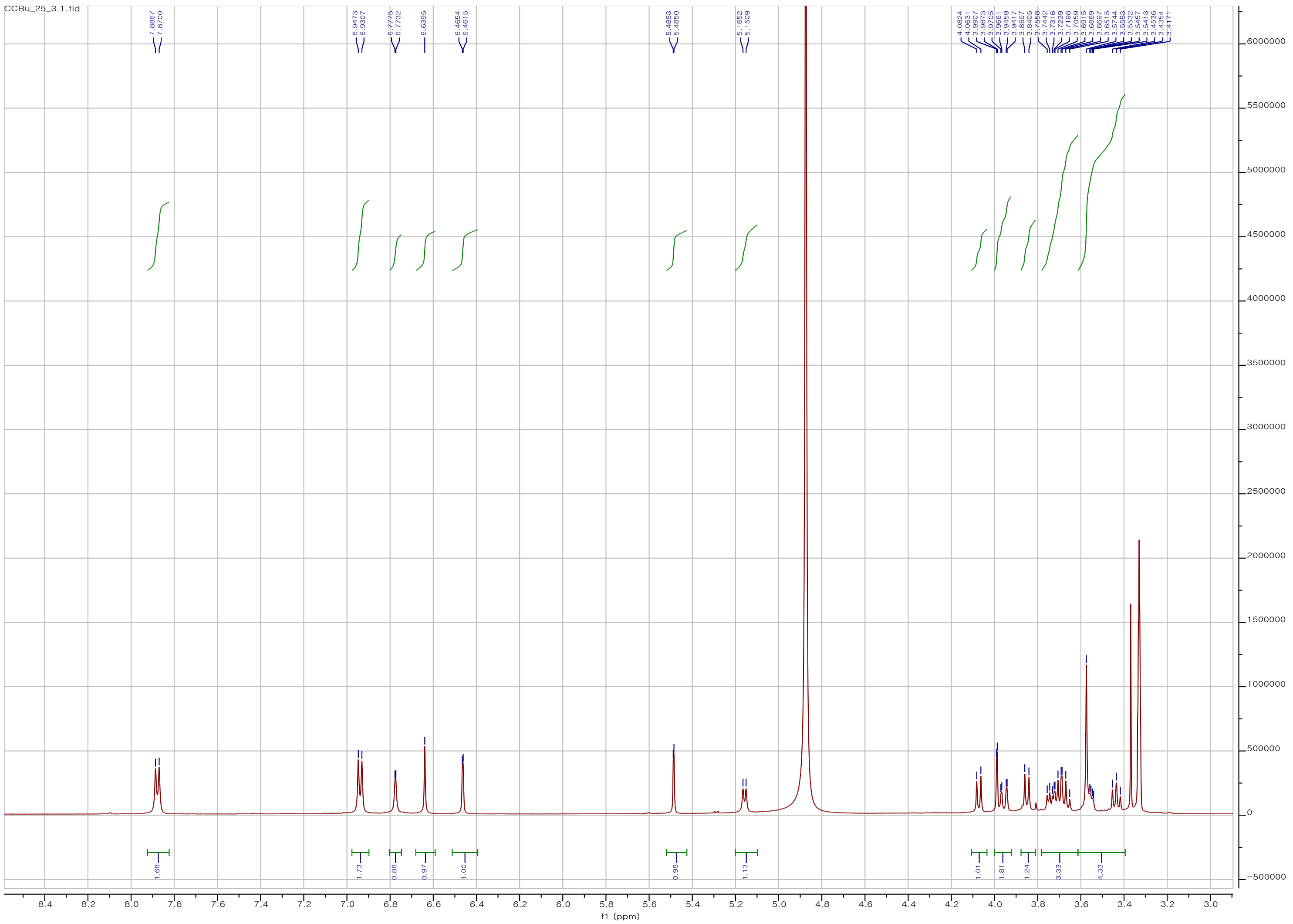

(A)

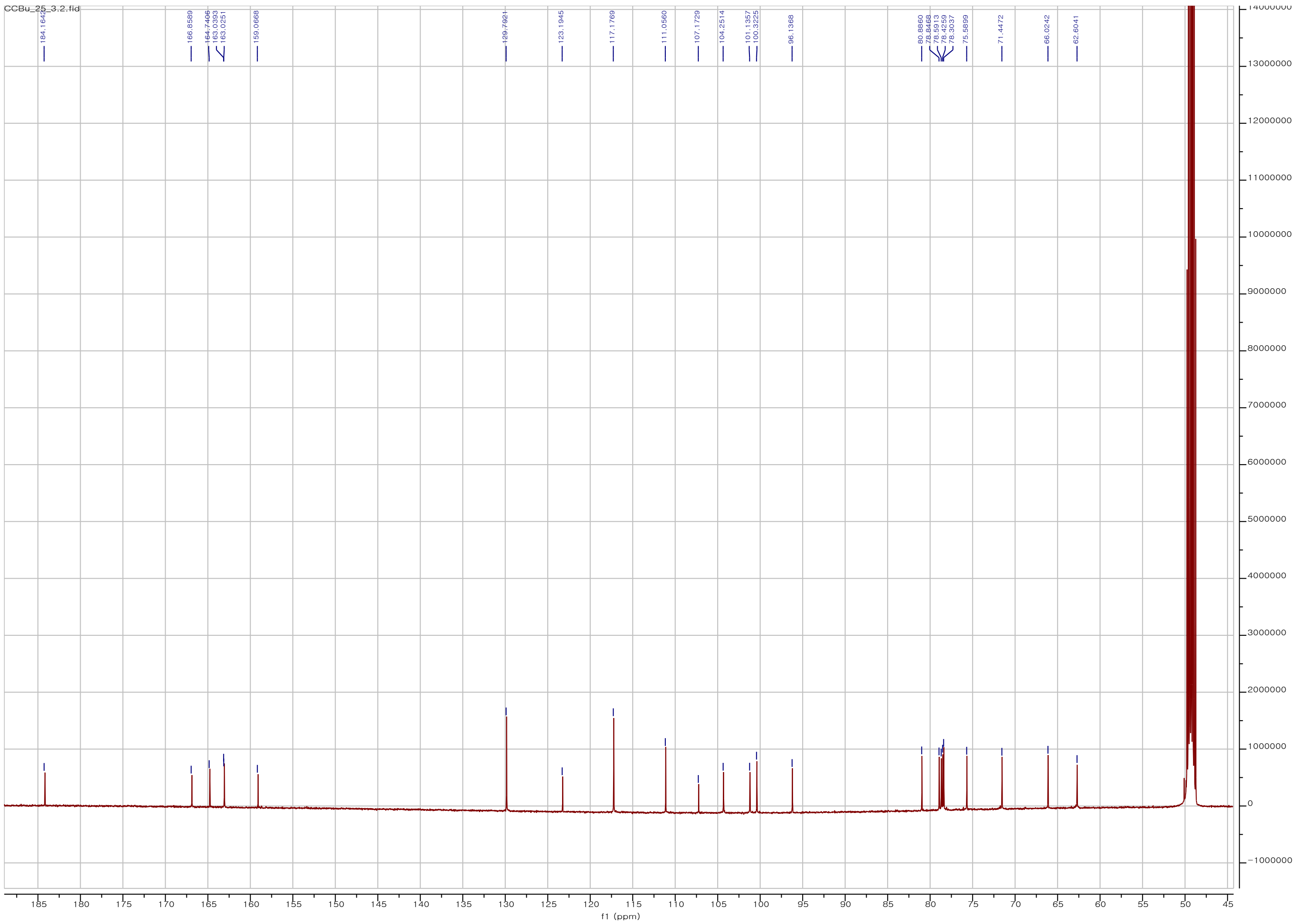

(B)

Figure S6. (A) ^{1}H-NMR spectrum of compound **6** in CH_3OH-d_4 at 500 MHz. (B) ^{13}C-NMR spectrum of compound **6** in CH_3OH-d_4 at 125 MHz.

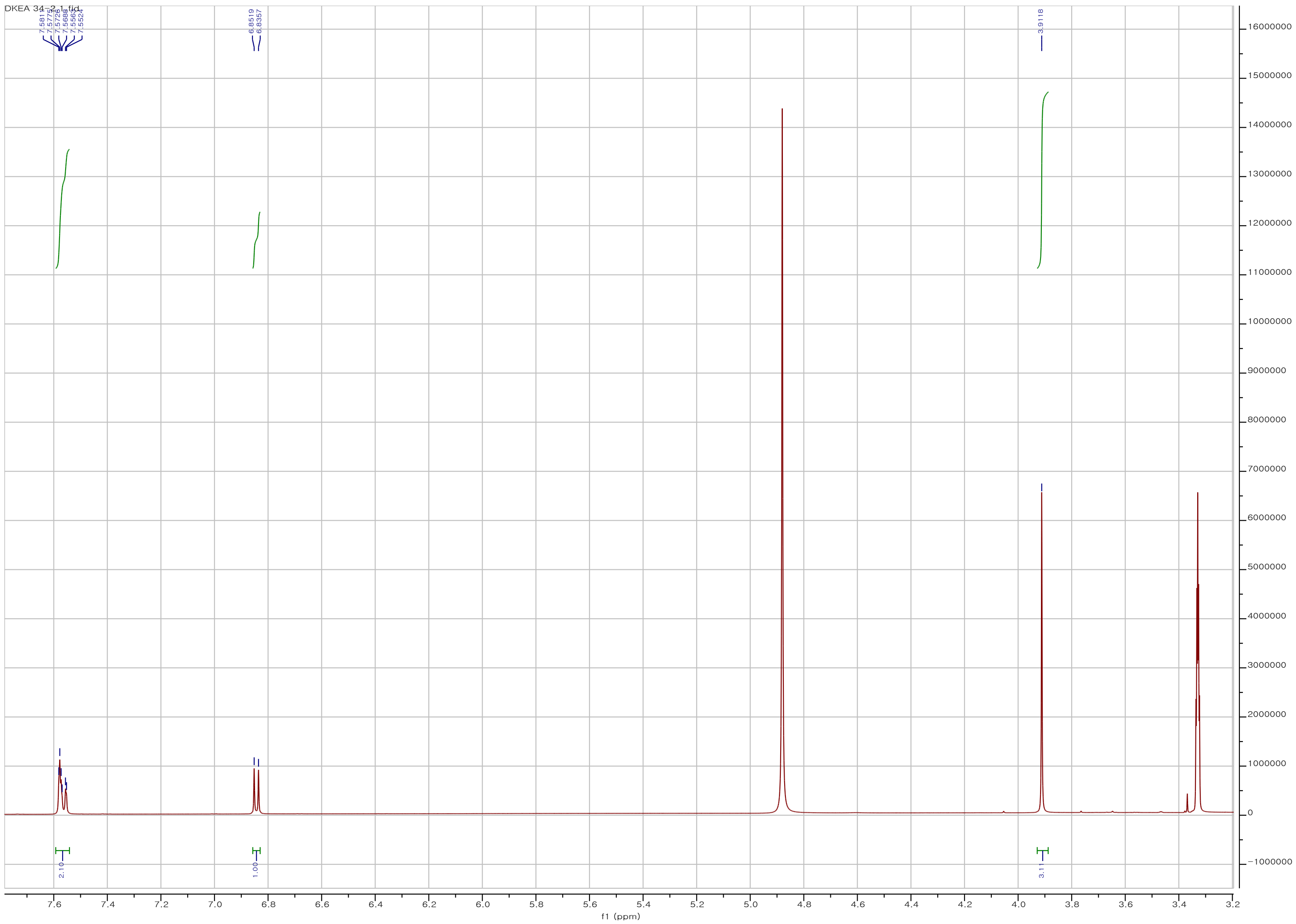
DKEA 34_2.1.fid
7.5817
7.5770
7.5726
7.5688
7.5564
7.5524
6.8519
6.8357
3.9118
2.10
1.00
3.11
f1 (ppm)

(A)

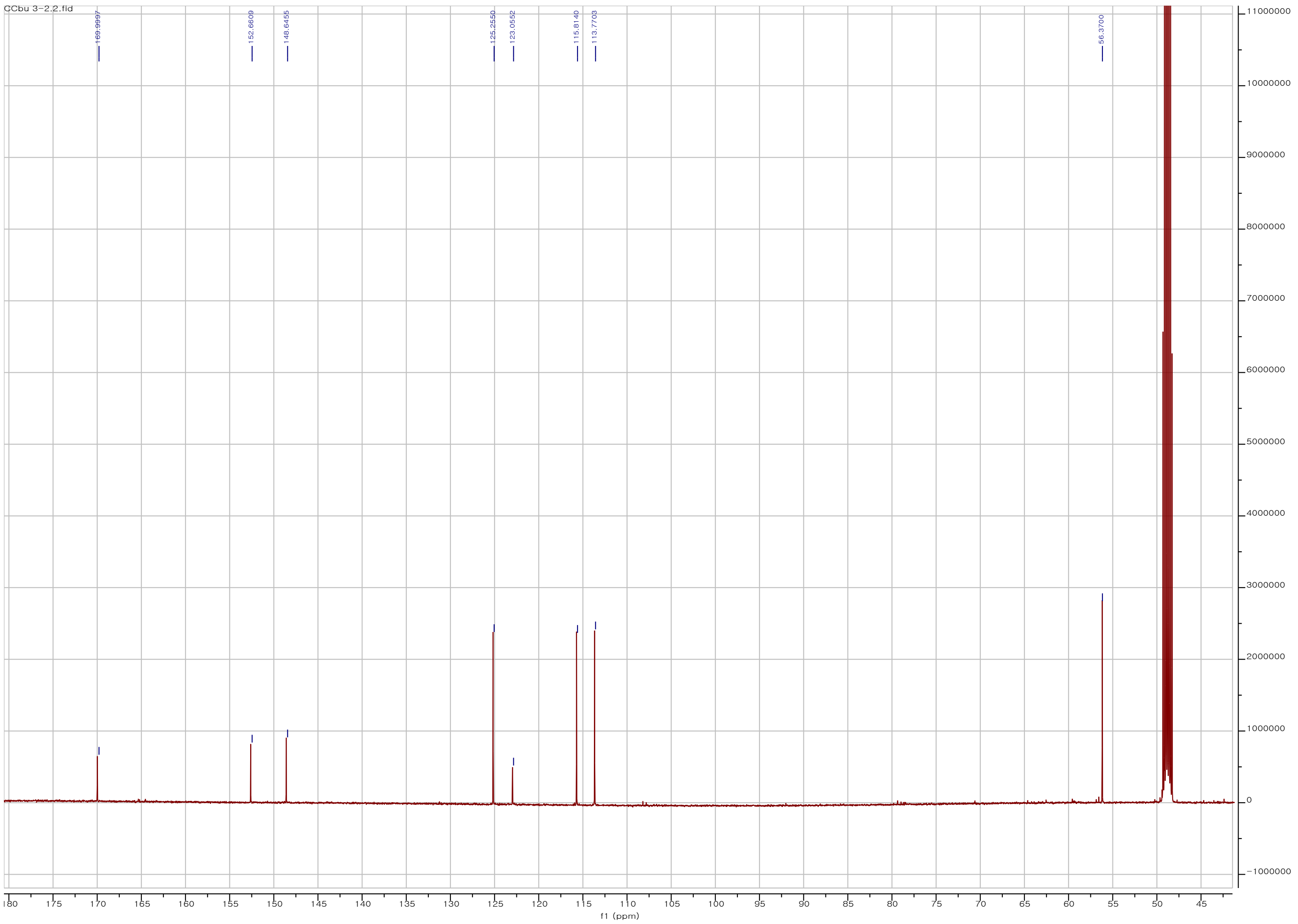

(B)

Figure S7. (A) ^{1}H-NMR spectrum of compound **7** in CH_3OH-*d*$_4$ at 500 MHz. (B) ^{13}C-NMR spectrum of compound **7** in CH_3OH-*d*$_4$ at 125 MHz.

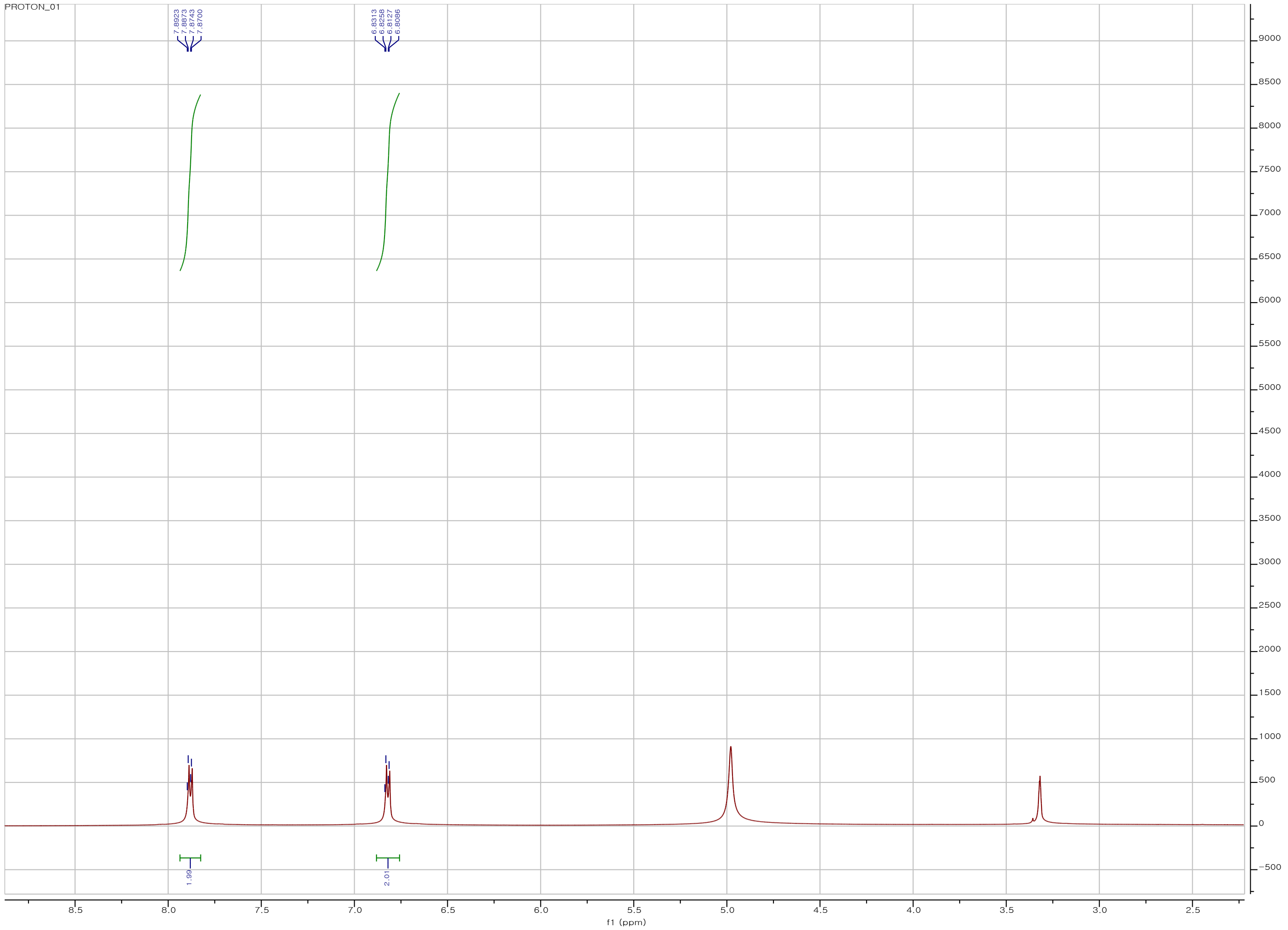
PROTON_01
7.8923
7.8873
7.8743
7.8700
6.8313
6.8258
6.8127
6.8086
1.99
2.01
f1 (ppm)
8.5
8.0
7.5
7.0
6.5
6.0
5.5
5.0
4.5
4.0
3.5
3.0
2.5
9000
8500
8000
7500
7000
6500
6000
5500
5000
4500
4000
3500
3000
2500
2000
1500
1000
500
0
-500

(A)

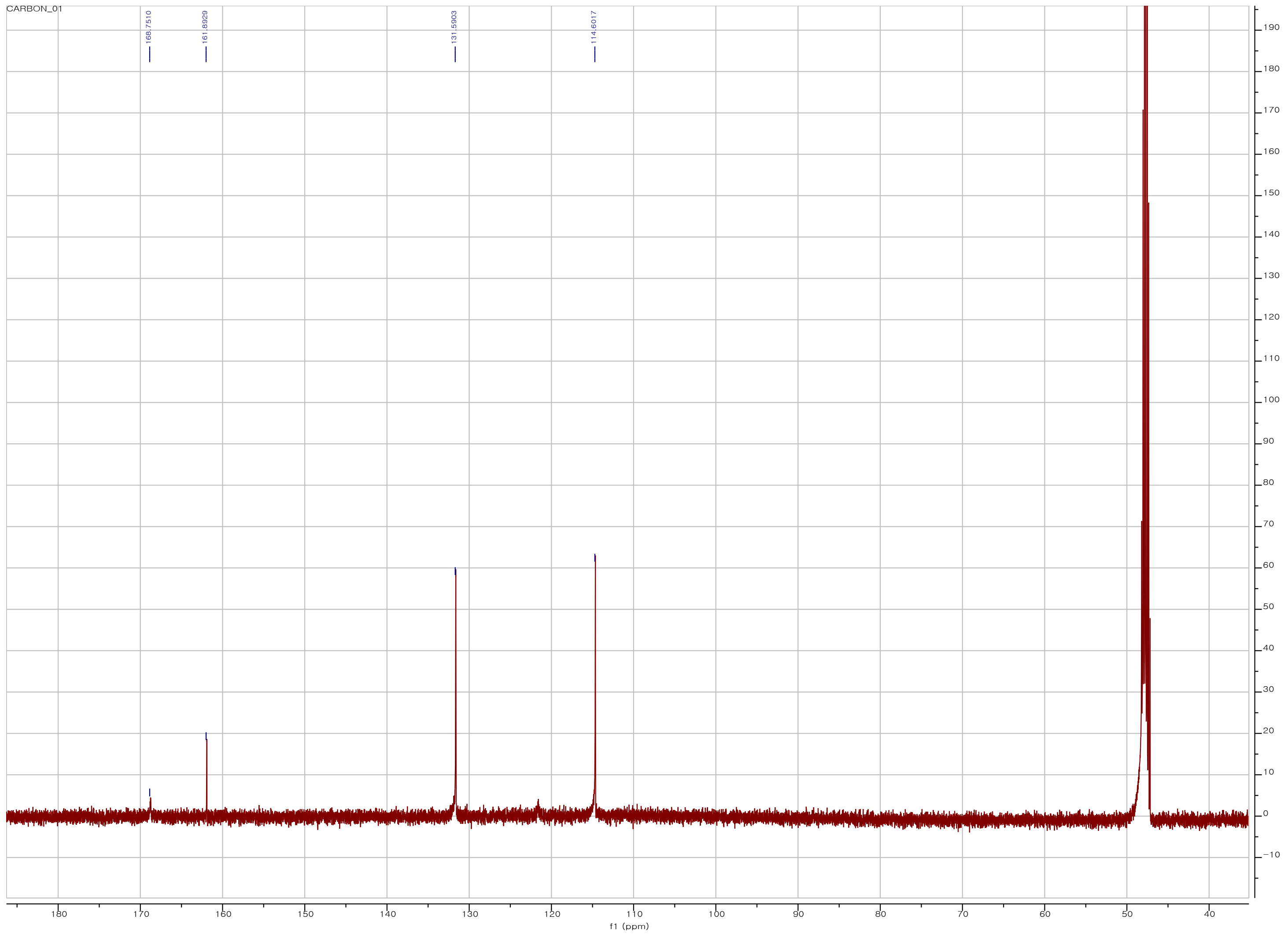

(B)

Figure S8. (A) ^{1}H-NMR spectrum of compound **8** in CH_3OH-d_4 at 500 MHz. (B) ^{13}C-NMR spectrum of compound **8** in CH_3OH-d_4 at 125 MHz.

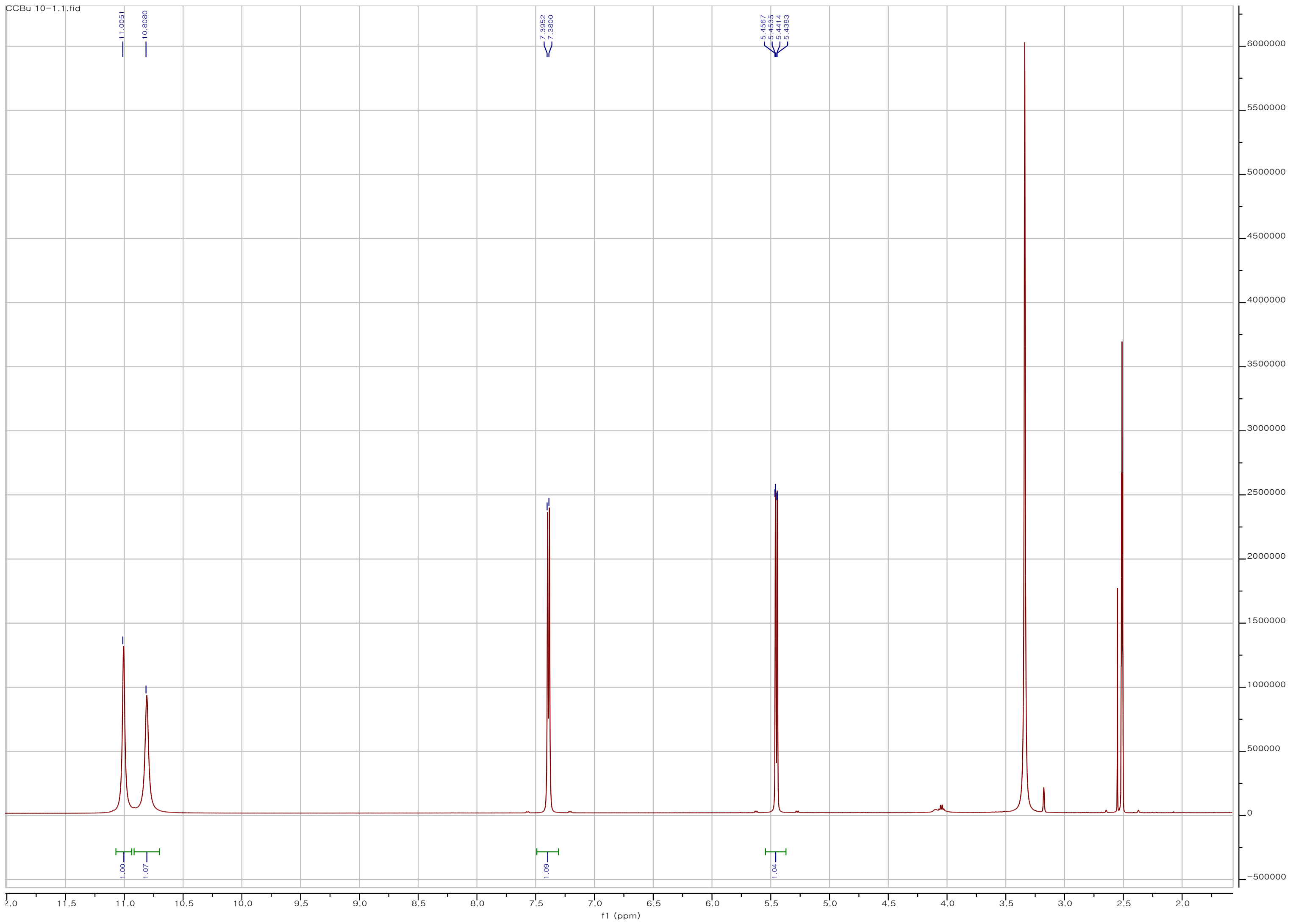
CCBu 10-1.1.fid
11.0051
10.8080
7.3952
7.3800
5.4567
5.4414
5.4383
1.00
1.07
1.09
1.04
f1 (ppm)

(A)

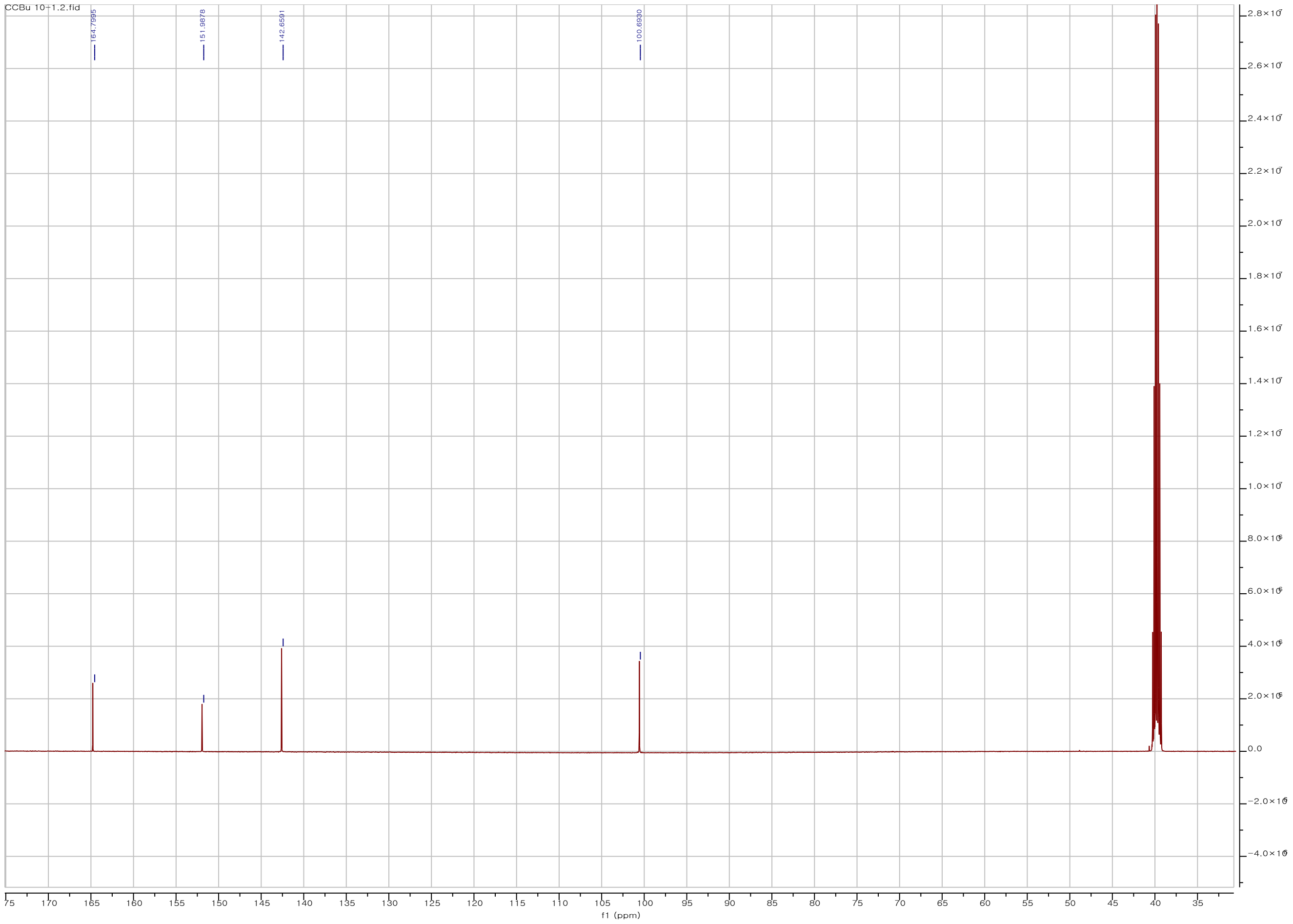

(B)

Figure S9. (A) ^{1}H-NMR spectrum of compound **9** in DMSO-d_6 at 500 MHz. (B) ^{13}C-NMR spectrum of compound **9** in DMSO-d_6 at 125 MHz.
